# AhR activation triggers the synergistic genotoxicity between benzo[a]pyrene and aflatoxin B1 in metabolic-competent HepaRP cells

**DOI:** 10.1007/s00204-026-04491-z

**Published:** 2026-07-14

**Authors:** Anis Messaoudi, Marie Garcia, Marc Audebert, Julien Vignard, Gladys Mirey

**Affiliations:** Toxalim, Université de Toulouse, INRAE, ENVT, EI-Purpan, Toulouse, France

**Keywords:** Mixture, BMC approach, Mode of action, CYP450 bioactivation, Genotoxicity, Synergistic interaction

## Abstract

Human dietary exposure involves multiple contaminants, raising concerns about “cocktail” effects ignored by single-compound risk assessment. Benzo[a]pyrene (BaP) and aflatoxin B1 (AFB1) are co-occurring food contaminants requiring cytochrome P450–mediated bioactivation. They both form DNA-reactive metabolites and are classified as genotoxic carcinogens. Here, BaP and AFB1 genotoxicity was evaluated individually and in binary combination in metabolically competent HepaRP cells using γH2AX as DNA damage biomarker. Concentrations were selected to reflect physiologically relevant levels (BaP: 0.125–10 µM; AFB1: 0.01–2 µM). Benchmark concentration (BMC) analysis with the PROAST covariate approach allowed deriving potency estimates, predicting mixture effects under dose addition hypothesis. Cells co-exposed to BaP-AFB1 presented a synergistic increase in γH2AX foci number compared to additive model predictions. Mechanistic investigations identified aryl hydrocarbon receptor (AhR) signaling as a key driver of this interaction. First, AhR pharmacological activation with β-naphthoflavone (BNF) recapitulated the effects of BaP, enhancing the genotoxicity induced by AFB1 exposure. In contrast AhR inhibition with CH223191 reduced DNA damage in cells treated with BaP, BaP-AFB1 or BNF-AFB1. Consistent with the metabolic interaction hypothesis, BaP or BNF alone or in mixture with AFB1 increased the expression and activity of CYP1 family enzymes, while CH223191 prevented these responses. Finally, non-genotoxic polycyclic aromatic hydrocarbons (PAHs) as fluoranthene and phenanthrene also enhanced AFB1 genotoxicity. Altogether, these results underline the importance of AhR-dependent metabolic activation in synergistic interaction within mixtures, supporting the necessity of mechanistic knowledge to improve risk assessment, notably for co-exposure to food contaminants.

**Supplementary Information:**

The online version contains supplementary material available at https://doi.org/10.1007/s00204-026-04491-z.

## Introduction

With the concept of exposome (Wild 2005), the challenge of food exposome also emerged and pointed out the necessity of studying mixtures (Tralau et al. 2021). Indeed, through their diet, consumers are chronically exposed to a wide range of contaminants that differ in origin, nature (organic or inorganic), chemical structure and toxicological properties (Duan et al. 2025). Among food contaminants, polycyclic aromatic hydrocarbons (PAHs) are persistent organic compounds of particular concern. PAHs are generated primarily as by-products, through incomplete combustion processes or via high-temperature food processing (Famiyeh et al. 2021). Food preparation methods such as grilling, smoking and charring significantly contribute to PAH formation (Sampaio et al. 2021). Benzo[a]pyrene (BaP), a prototypical and particularly hazardous PAH, is classified as a Group 1 human carcinogen by the International Agency for Research on Cancer (IARC 2012). The European Food Safety Authority (EFSA) estimates that the European population is exposed through diet to 3.9–6.5 ng/kg body weight per day of BaP (EFSA 2008). A benchmark dose lower confidence limit (BMDL_10_) of 0.07 mg/kg body weight per day has been derived from tumor incidence observed in long‑term animal carcinogenicity studies and is used as the point of departure (POD) for dietary cancer risk assessments of PAHs (EFSA 2008). Following intestinal absorption and hepatic transport, BaP undergoes metabolic activation by phase I enzymes, mainly cytochrome P450 1A1 (CYP1A1) and 1B1 (CYP1B1), forming the ultimate carcinogen B[a]P-7,8-dihydrodiol-9,10-epoxide (BPDE) (Šulc et al. 2016). This highly reactive metabolite binds covalently to guanine bases in DNA to form genotoxic adducts that contribute to carcinogenesis (Tretyakova et al. 2002; Poirier 2016). Beyond PAHs, humans are also exposed to mycotoxins, with aflatoxin B1 (AFB1), produced by *Aspergillus flavus and Aspergillus parasiticus*, being the most potent natural carcinogen identified so far (Murray et al. 2016). AFB1 contaminates staple foods worldwide, especially in tropical and subtropical regions that favor fungal growth with AFB1 detected in 38.5% of rice samples in Asia (Ali 2019). Contamination is often higher in Africa, with all rice samples containing AFB1 in Nigeria (4.1–309 µg/kg) or Ivory Coast (1.5–10 µg/kg) (Ali 2019), highlighting significant dietary exposure frequently above regulatory thresholds (Jallow et al. 2021). Climate change is expected to markedly increase AFB1 occurrence in Europe, making it an emerging and significant food safety challenge (Battilani et al. 2016; Leggieri et al. 2021). EFSA CONTAM Panel estimates dietary exposure to AFB1 in Europe at 0.1–0.5 ng/kg body weight per day, and a BMDL_10_ increase in hepatocellular carcinoma incidence was set at 0.4 µg/kg body weight per day based on long‑term animal carcinogenicity data (EFSA 2020). AFB1 poses a serious health risk due to its cytotoxic, genotoxic, and carcinogenic properties (Mahato et al. 2019). Indeed, after absorption in the small intestine, AFB1 is metabolized primarily in the liver by cytochrome P450 enzymes (CYP1A2, CYP3A4), producing the highly reactive AFB1-8,9-epoxide (AFBO) and AFM1. AFBO forms adducts with DNA, promoting mutagenesis and hepatocellular carcinoma (Smela et al. 2002). AFB1 is thus classified as a Group 1 human carcinogen (IARC 2012).

Traditionally, toxicological risk assessment evaluates chemicals individually. However, this approach may underestimate risk by ignoring cocktail effects, i.e., interactions arising when substances co-occur. In recent years, this issue has received increasing attention, prompting the development of cumulative risk assessment frameworks (Boobis et al. 2008; EFSA 2019). To evaluate the toxicity and risk of chemical mixtures with similar modes of action, the concept of concentration addition, or dose addition, was introduced. In this framework, risk is determined not only by toxicity and exposure, as with single chemicals, but also by the number of components in the mixture, which can influence the overall effect. In food toxicology, contaminants sharing the same mode of action are generally assumed to produce additive effects, as commonly applied to PAHs (EFSA 2019). However, this assumption may overlook cellular processes such as receptor interactions, enzyme modulation, or other intracellular mechanisms, potentially leading to under- or overestimation of actual risk. These considerations underscore the importance of mixture-based assessments that account for complex interactions. Regulatory approaches such as the Mixture Allocation Factor (MAF) have been proposed to address cumulative exposure. The MAF assigns to each substance a fraction of the total tolerable risk, ensuring that combined exposures remain within safe limits and strengthening EU chemical risk management (Backhaus et al. 2025).

Biological effects of mixtures can be characterized by examining the interactions between their components, which may be additive, antagonistic, or synergistic (OECD 2018; Lasch et al. 2020). The benchmark dose/concentration (BMD/BMC) approach has been proposed, particularly the BMC covariate method, which is an advanced statistical approach used in concentration-response modeling, especially for risk assessment (EFSA 2019; Sanders et al. 2025; Slob et al. 2025). This method involves incorporating independent variables, other than concentration, known as covariates (e.g., the different chemicals) that may influence the biological response, allowing the simultaneous estimation of BMCs for the various substances (EFSA 2022). When chemicals within a mixture interact, this method enables a more accurate assessment of their combined effects and relative potencies (Wills et al. 2016; Slob et al. 2025).

Dietary co-exposure to AFB1 and BaP illustrates the public health relevance of mixture effects. These compounds frequently co-occur in foods such as edible oils, cereals, and smoked products (Wang et al. 2024b; Huang et al. 2025), and their combined exposure may produce additive or synergistic toxic effects. AFB1 can be bioactivated by human CYP1A1 (Wang et al. 2024a), an enzyme regulated by the aryl hydrocarbon receptor (AhR) pathway (Ma 2001). Because BaP is a potent AhR agonist (Matsunawa et al. 2009), it may induce CYP1A1, CYP1B1 and CYP1A2 upregulation, enhancing AFB1 activation and potentially amplifying its genotoxicity.

In this study, we aimed to apply the BMC covariate approach and assess the combined genotoxic effects of BaP and AFB1, two compounds with similar genotoxic mode of action. Nine concentrations of BaP (0.125–15 µM) or AFB1 (0.01–2 µM) were tested in metabolically competent HepaRG-derived hepatocytes (HepaRP) that expresses major phase I and phase II drug-metabolizing enzymes as well as key nuclear receptors and in contrast to parental HepaRG cultures, HepaRP consist exclusively of hepatocytes (Brun et al. 2023). In addition, differentiated HepaRP consist exclusively of hepatocytes, allowing high-content imaging analysis of DNA damage through the γH2AX biomarker (Kuo and Yang 2008; Kopp et al. 2019). Concentration–response data were then used to derive BMC values at multiple benchmark response (BMR) levels, reflecting physiologically relevant exposure in line with EFSA safety thresholds. Mixtures were then assessed using different BaP-AFB1 ratios, revealing synergistic genotoxic effects. Mechanistic investigations with AhR pharmacological modulators showed that AhR activation triggered AFB1-induced DNA damage. Moreover, CYP450 induction as well as elevated CYP1A1/1B1 activities, impeded by AhR antagonist, underlined that BaP-AFB1 synergistic genotoxicity is driven by AhR-mediated metabolic modulation. As non-genotoxic polycyclic aromatic hydrocarbons (PAHs) like fluoranthene or phenanthrene enhanced AFB1 genotoxicity, this underlines the importance of AhR-dependent metabolic activation in synergistic interaction within mixtures and the necessity of mechanistic knowledge to improve risk assessment.

## Materials and methods

### Chemicals

BaP (CAS 50-32-8), AFB1 (CAS 1162-65-8), Phenanthrene (CAS 85-01-8) and CH223191 (CAS 301326-22-7) were purchased from Sigma-Aldrich (France). BNF (CAS 6051-87-2) was purchased from thermo scientific (Germany). Fluoranthene (CAS 206-44-0) was purchased from Fluka AG (Saint-Gallen, Suisse). All chemicals were dissolved in Dimethyl sulfoxide (DMSO) (Sigma-Aldrich, St. louis, USA).

## Cell Culture

HepaRG-derived HepaRP cells (Brun et al. 2023) were obtained from Biopredic International (#HPR132004, Saint Grégoire, France) and cultured according to supplier recommendations, under Material Transfer Agreement. Briefly, cells were cultured in a T75 flask in proliferation medium (ADD310008, Biopredic-International) for two weeks at 37 °C in a humidified atmosphere with 5% CO_2_ until confluence. Then, the proliferation medium was replaced with differentiation medium (ADD320008, Biopredic International) supplemented with DMSO for an additional two weeks. The differentiation medium was renewed every 2–3 days with increased concentrations of DMSO (0%; 0.2%; 0.4%; 0.8%; 1.2%). Then, differentiated cells were seeded in 96-well plates at a density of 8 × 10^4^ cells/well for immunofluorescence and CYP1A1/1B1 activity assay and in 6-well plates at a density of 1.5 × 10^6^ cells/well for western blot assay. Three days after seeding, the differentiation medium was replaced, and 24-hours later, the cells were treated for further analyzes.

## Genotoxicity analysis

Genotoxicity assessment was performed as described before (Recoules et al. 2025). Briefly, after treatment, cells were fixed for 20 min in 4% paraformaldehyde, then washed with phosphate-buffered saline (PBS) and permeabilized for 10 min in 0.5% Triton X-100. Cells were subsequently blocked with 3% BSA and 0.1% IGEPAL. Cells were then incubated for 2- hours at room temperature in blocking solution containing the primary antibody against phospho-Histone H2AX (Ser139), referred to as γH2AX (Merck/Millipore 05-636-1, 1:1000). After incubation, primary antibody was removed and cells were washed three times with PBS containing 0.1% IGEPAL before incubation with the secondary antibody (Alexa Fluor 546 Goat anti-mouse, 1:1000) for 30 min at room temperature. DNA was stained with 4’,6-diamidino-2-phenylindole (DAPI) at 500 nM. Cells were analyzed using the ImageXpress Micro Confocal High-Content Imaging System (Molecular Devices) with a 40X water-immersion objective. The number of γH2AX foci was quantified using the MetaXpress software in at least 2,000 cells. Viability was assessed by counting the number of DAPI-stained nuclei on 4X images (encompassing approximately 50% of the well and a minimum of 40,000 nuclei in DMSO control condition) using the MetaXpress software. Relative Cell Count (RCC) was then calculated by normalizing the number of nuclei in each well to the mean nuclei count of the DMSO control, which was set to 100%. Results from all treatment conditions are therefore expressed as a percentage relative to the DMSO control (RCC%).

## Protein level analysis

Four days after seeding in 6-well plates, cells were first pre-treated with CH223191 (10µM) for 2 h to block AhR signaling when required. Cells were then treated with the compounds of interest for 8 h, either individually (BaP 5.231µM; AFB1 0.301µM) or with a 1:1 mixture containing 50% of each compound’s BMC. As positive control for AhR activation, cells were treated 8 h with BNF (1µM), either alone or in combination with AFB1 (0.301 µM). When needed, CH223191 was present throughout the pre-incubation and treatment phases, ensuring continuous inhibition of AhR signaling. Cells were then washed twice with PBS, lysed in Laemmli buffer, sonicated using a VibraCell sonicator (Bioblock Scientific), and heated at 95 °C for 5 min. Protein samples (25µL) were separated on 10% TGX Stain-Free polyacrylamide gels (Bio-Rad), and total protein content was visualized using Stain-Free technology with a ChemiDoc XRS imaging system (Bio-Rad). Proteins were subsequently transferred to nitrocellulose membranes using the Trans-Blot Turbo system (Bio-Rad) and subjected to western blot analysis. After blocking, membranes were incubated overnight at 4 °C with primary antibodies directed against CYP1A2 (1:250; D15, Santa Cruz Biotechnology), CYP1B1 (1:5000; J21C16, Selleckchem), or GAPDH (1:10000; GTX100118, GeneTex). Appropriate HRP-conjugated secondary anti-mouse or anti-rabbit antibodies (1:50000; Jackson ImmunoResearch Laboratories) were applied for 1-hour at room temperature. Protein bands were detected using enhanced chemiluminescence (ECL, Bio-Rad), imaged with the ChemiDoc XRS system and analyzed with the Image Lab software.

## CYP activity measurement

CYP1A1/1B1 enzyme activities were measured using the P450-Glo™ Luciferin-CEE assay (Promega) according to the manufacturer’s protocol. After a 2-hour pre-treatment with CH223191 (10µM) followed by a 8-hour treatment with BaP (5.231µM) and AFB1 (0.301µM), either individually or as a 1:1 mixture ratio (CH223191 was present throughout pre-incubation and treatment phases when needed), the medium was replaced by DMSO-free culture medium containing pro-luciferin, transformed by CYP450 enzymes into luciferin. After 3 h of incubation at 37 °C, an equal amount of luciferin detection reagent was added, and luminescence proportional to CYP450 activities was read using a Spark multimode microplate reader (Tecan, Lyon, France). The signals were normalized with the average signals of vehicle-treated cells.

### Benchmark concentration (BMC) covariate method for mixture studies

A BMC covariate approach was applied to binary food-contaminant mixtures (Sanders et al. 2025). First, individual concentration-response data (γH2AX foci number per nucleus) were generated for each compound using at least nine concentrations spanning low to high effect levels. Second, BMCs were derived at multiple benchmark response (BMR) levels (corresponding to 300%, and 400% increases over control using the PROAST web tool version 71.1 (https://proastweb.rivm.nl/) with covariate analysis. Third, mixtures were formulated assuming concentration additivity, preparing three ratios per BMR level (1:1, 3:1, 1:3) **(**Table 1**)** to assess equipotent and non-equipotent combinations. Fourth, all mixtures were tested using the same assay conditions as the individual compounds. Finally, mixture responses were modeled in RStudio (using the PROAST dose-addition model); accordance with the predicted curve indicates additivity, while deviations above or below the expected response suggest synergism or antagonism, respectively.

**Table 1:** Benzo[a]pyrene (BaP) and aflatoxin B1 (AFB1) calculated concentrations for the three mixture ratios (3:1, 1:1, 1:3), including single-compound concentrations (1:0 for BaP and 0:1 for AFB1) across different benchmark responses (BMRs)

Concentration of BaP and AFB1 within mixtures (µM)				
	BMR 300%		BMR 400%	
	BaP	AFB1	BaP	AFB1
***1:0 (BMC)***	*5.231*	*0*	*8.283*	*0*
***3:1***	*3.923*	*0.075*	*6.212*	*0.119*
***1:1***	*2.615*	*0.150*	*4.141*	*0.238*
***1:3***	*1.307*	*0.225*	*2.070*	*0.357*
***0:1 (BMC)***	*0*	*0.301*	*0*	*0.476*

### Statistical analysis

Data are presented as mean with standard error of the mean (SEM) in at least three independent experiments. Statistical analyses were performed using GraphPad Prism 10.3.1. Group comparisons were made using one-way or two-way ANOVA, as appropriate. Significance levels were defined as *p* < 0.05 (*), *p* < 0.01 (**).

## Results

### BaP or AFB1 exposure increases DNA damage in HepaRP cells in a concentration-dependent manner

**Fig. 1:**
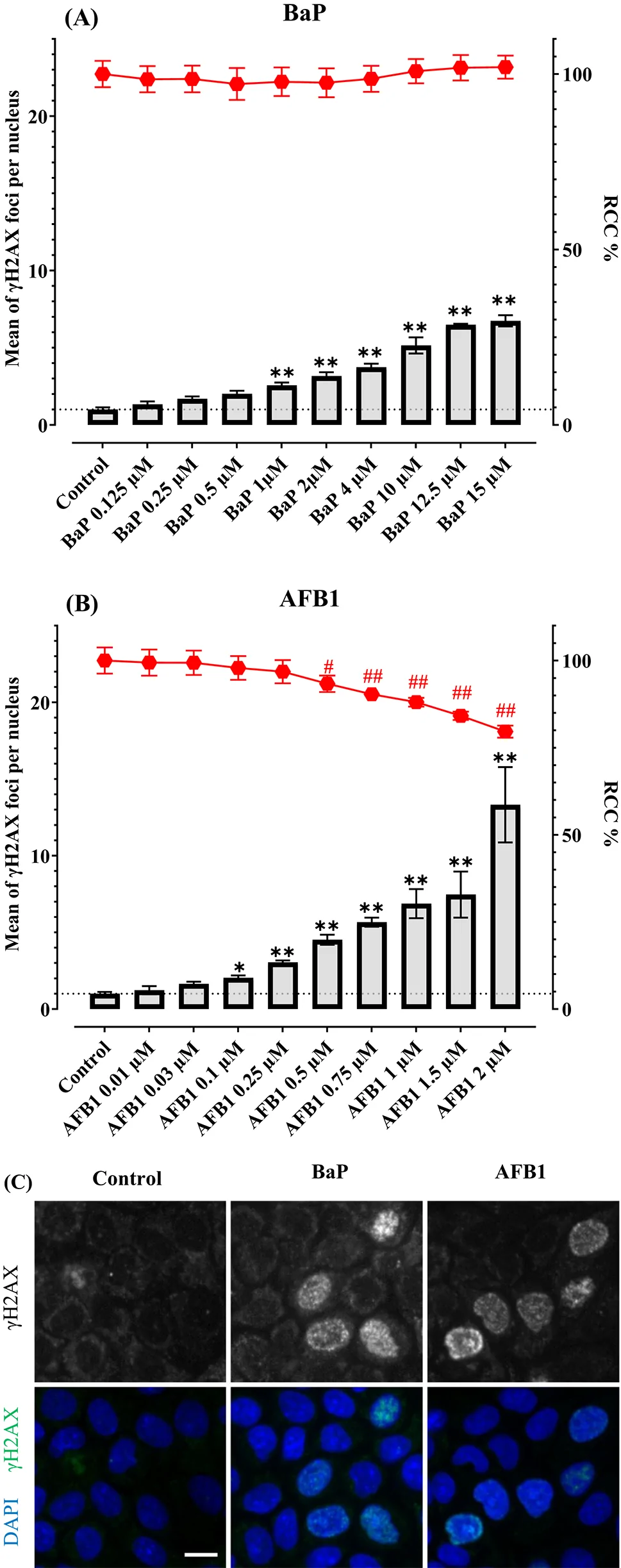
γH2AX immunofluorescence analysis of HepaRP cells exposed for 24 h to benzo[a]pyrene (BaP) or aflatoxin B1 (AFB1). The number of γH2AX foci per nucleus observed after 24 h exposure to BaP (**A**) or AFB1 (**B**) are presented. Bars show the mean number of γH2AX foci per nucleus ± SEM (*n* = 3), normalized to DMSO control and presented as fold change. Statistical comparisons to DMSO control were performed using one-way ANOVA: *p* < 0.05 (*), *p* < 0.01 (**). Dots indicate the mean percentage of relative cell count (RCC%) ± SEM (*n* = 4), calculated from the number of counted nuclei compared to untreated controls. Statistical comparisons to DMSO control for cell counts were performed using one-way ANOVA: *p* < 0.05 (#), *p* < 0.01 (##). Representative images are shown in (C), with staining γH2AX foci in grey or the merge image with DAPI and γH2AX staining. Scale bar = 20 μm

In order to evaluate the genotoxic effect of BaP or AFB1, the number of γH2AX foci per nucleus was evaluated in differentiated HepaRP cells exposed to increasing concentrations of BaP **(**Fig. 1A**)** and AFB1 **(**Fig. 1B**)**, together with the relative cell count (RCC%). Both compounds induced a significant concentration-dependent increase in γH2AX foci formation, underlining their genotoxic properties. In BaP-treated cells, a significant increase in the mean number of γH2AX foci was observed starting at 1 µM, corresponding to an approximately 2.5-fold increase compared with control. At the highest tested concentration (15 µM), γH2AX foci levels reached a 6.7-fold increase relative to the DMSO control. No significant decrease in RCC% was observed across all tested BaP concentrations, indicating the absence of cytotoxicity under these conditions. In contrast, AFB1 exposure resulted in a marked induction of γH2AX foci at lower concentrations. A significant increase was detected at 0.1 µM, with an approximately 2-fold change compared with negative control, reaching up to a 13-fold increase at the highest concentration. This was accompanied by a significant reduction in RCC%, with cell viability decreasing to 93% at 0.5 µM and to approximately 79% at 2 µM, indicating concentration-dependent cytotoxicity. Concentration-response data were analyzed using the covariate approach implemented in PROAST (Supplementary Information 1), at benchmark response (BMR) levels of 300% and 400%, and benchmark concentrations (BMCs) were derived for both compounds. The black curve represents the experimental concentration-response data of BaP while the red curve represents the experimental concentration-response data of AFB1.

### Combined exposure to BaP and AFB1 leads to a synergistic increase of DNA damage

Based on the BMC values calculated for each compound, mixtures of BaP and AFB1 were subsequently prepared and tested at different ratios. For each BMR, BaP and AFB1 were evaluated individually (1:0 and 0:1 ratio, corresponding to their respective BMCs) as well as in mixtures with equipotent (1:1) and non-equipotent ratios (3:1 and 1:3), in order to assess the relative contribution of each compound to the observed effect **(**Table 1**)**.

The mean number of γH2AX foci per nucleus and the distribution of cells according to γH2AX foci number (0–1, 2–5, or > 5 foci) were evaluated in BaP and AFB1 treated cells, either individually or in mixtures, at the different tested BMR levels.

**Fig. 2:**
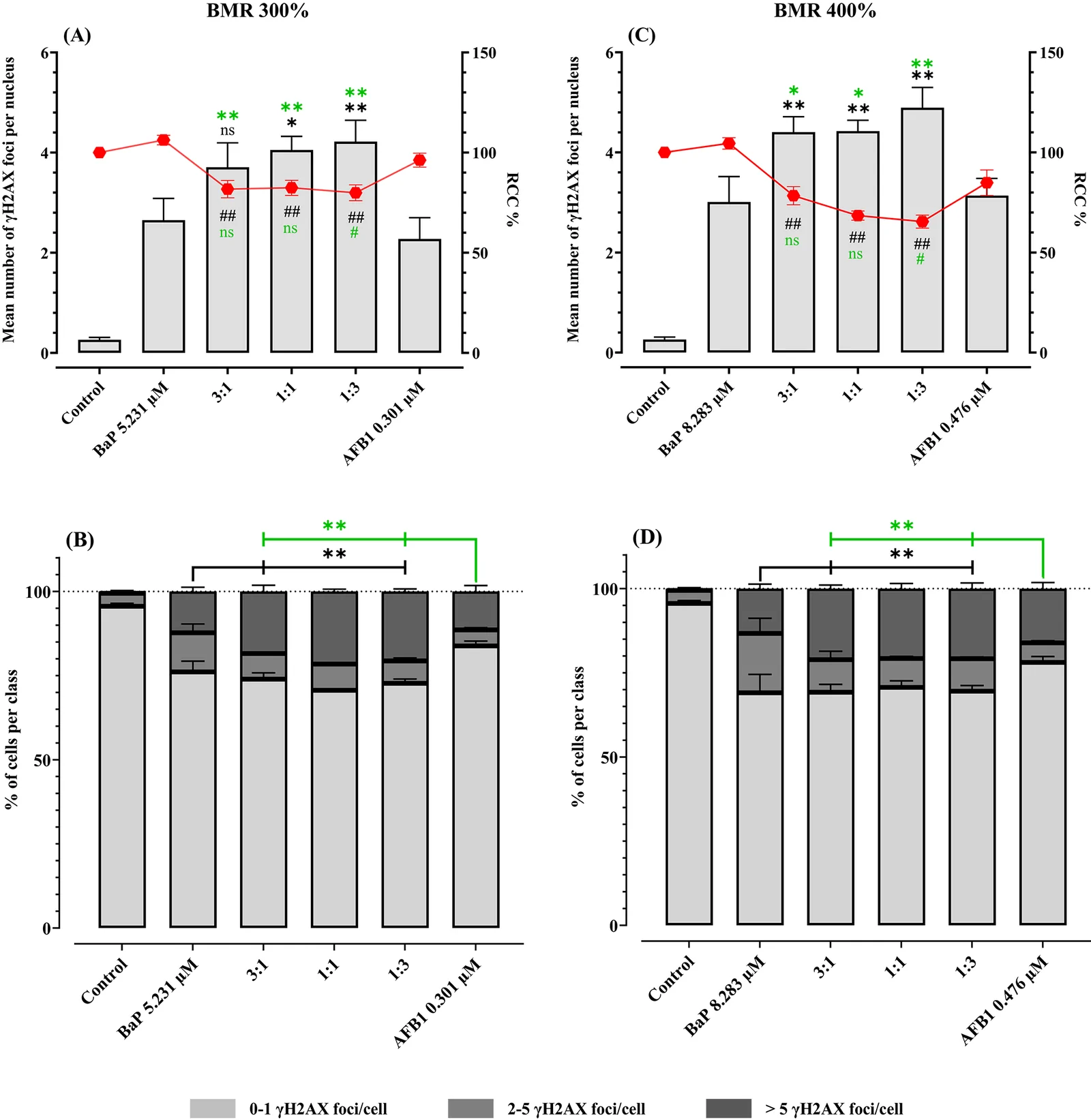
Mean number of γH2AX foci per nucleus and cell classification based on foci number after BaP and AFB1 treatment at BMR300% or BMR400%. The mean number of γH2AX foci after 24 h treatment with BaP and AFB1, applied alone or in mixtures, and the mean percentage of relative cell count (RCC%) are presented at BMR300% and BMR400% (2-A and 2-C respectively). Bars represent mean ± SEM (*n* = 3), and dots represent RCC% ± SEM (*n* = 3). The percentage of cells presenting 0–1, 2–5 or > 5 foci of γH2AX for the different mixture ratios are presented for BMR300% and BMR400% (2-B and 2-D respectively). Statistical analyses were performed by one-way ANOVA (for γH2AX foci mean number) or two-way ANOVA (for % of cell classes) comparing BaP alone with mixtures and AFB1 alone with mixtures. Comparisons of BaP with mixtures are indicated by black symbols, whereas comparisons of AFB1 with mixtures are indicated by green symbols (* for mean γH2AX foci, **#** for RCC%). *p* < 0.05 (* or #) and *p* < 0.01 (** or ##)

At BMR 300%, as expected, cells treated with BaP or AFB1 alone exhibited same mean γH2AX foci numbers per nucleus (approximately 2.5) (Fig. 2A). On the opposite, mixture treatments significantly increased γH2AX foci formation, with mean values rising from approximately 3.7 foci per nucleus for the 3:1 ratio to 4.2 foci per nucleus for the 1:3 ratio. Consistent with this, the proportion of cells containing more than five γH2AX foci increased from near 12% in BaP or AFB1 treated cells to 19% and 22% in the 3:1 and 1:3 mixtures, respectively (Fig. 2B). These increased DNA damages were associated with a decrease in RCC in the three mixtures tested, while BaP and AFB1 alone did not significantly affect cell viability.

At BMR 400%, a comparable pattern was observed. BaP and AFB1 alone induced mean γH2AX foci numbers of approximately 3 per nucleus, whereas mixture exposures resulted in significantly higher values, ranging from approximately 4.4 foci per nucleus at the 3:1 ratio to 4.9 foci per nucleus at the 1:3 ratio (Fig. 2C). The proportion of cells with more than five γH2AX foci increased from 15% for individual compounds to 22% in mixture-treated cells (Fig. 2D). Concomitantly, RCC values declined from approximately 103% and 84% for BaP and AFB1 alone to 78% and 65% in the 3:1 and 1:3 mixtures, respectively.

**Fig. 3:**
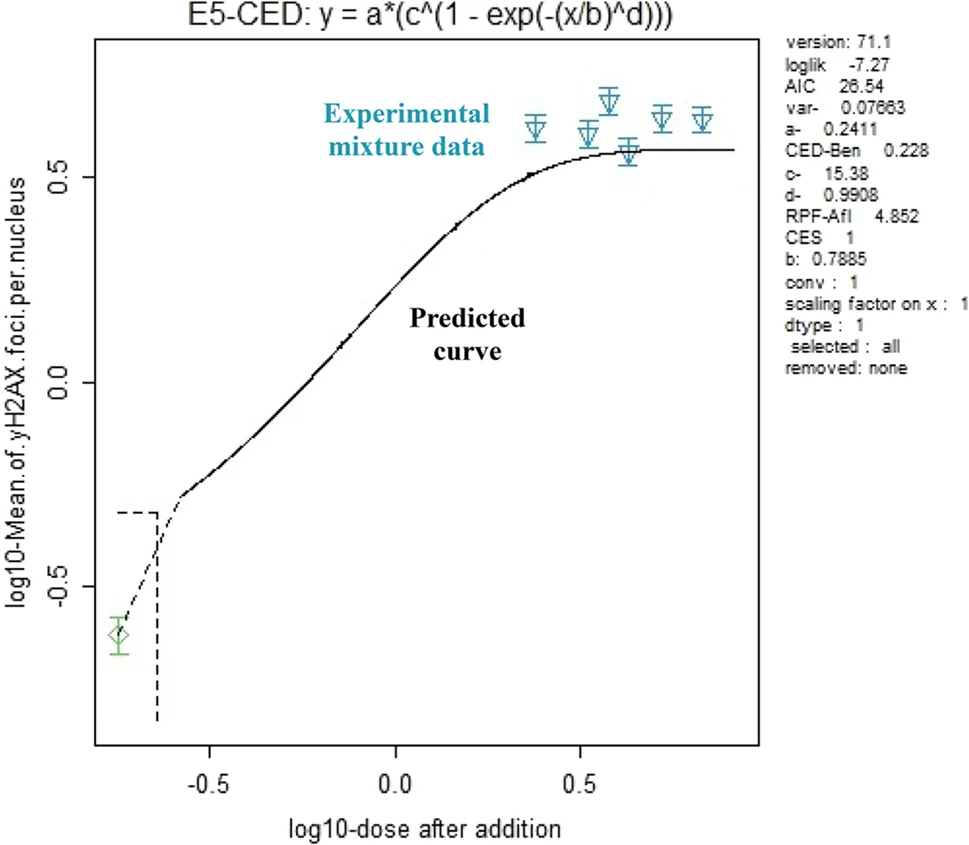
Comparison of BaP and AFB1 mixture responses to additive model. BaP and AFB1 responses were measured by γH2AX immunofluorescence (mean number of γH2AX foci), modeled in R using dose-addition model 15. Black curve represents predicted concentration-response based on additivity. Mixtures responses are presented (dark blue downward triangles) at BMR300% and BMR400% (from left to right) with their corresponding 95% confidence intervals

To evaluate the interaction between BaP and AFB1 through the principle of additivity, the experimental mixture data were analyzed using the PROAST dose-addition model in R. As shown in Fig. 3, the experimental mixture responses for γH2AX foci induction were plotted against the predicted concentration–response curve derived under the assumption of additivity. The experimental points obtained with the mixtures displayed clear up- and leftward deviations from the predicted additive curve, indicating synergistic interactions within the mixtures.

In conclusion, our findings indicate that combined exposure to BaP and AFB1 produces a synergistic enhancement of genotoxicity compared to the single-compound exposure, supporting the presence of a biological interaction between these compounds at the cellular level.

### Metabolic activation loop after BaP-AFB1 co-exposure induces synergistic genotoxic effects

As our results support the synergistic interaction between BaP and AFB1, we aimed to elucidate the biological mechanistic basis underlying this effect. We hypothesized that BaP-mediated AhR activation enhances CYP1A1 and CYP1A2 expression and thereby increases the metabolic activation of AFB1, ultimately contributing to the observed increased genotoxicity. To test this hypothesis and evaluate the involvement of the AhR pathway, mechanistic modulators, including a non-genotoxic AhR agonist (BNF) and a selective AhR antagonist (CH223191), were used.

**Fig. 4:**
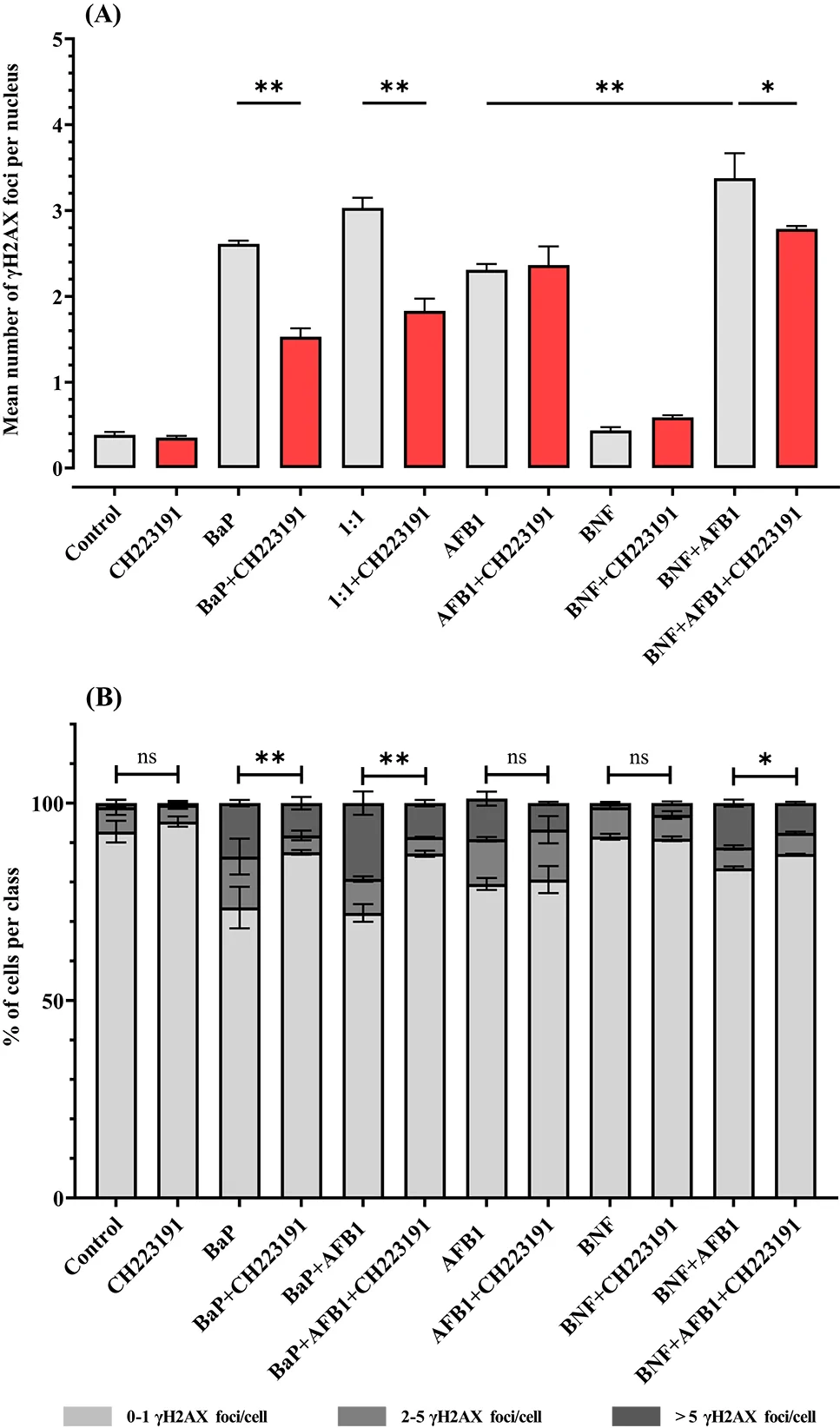
Mechanistic analysis with AhR pharmacological modulators. Mean number of γH2AX foci per nucleus (4-**A**) and distribution of cell classes based on γH2AX foci number (4-**B**) following 24-hour treatment with BaP and AFB1, either alone or in a 1:1 ratio combination at a BMR300%, as well as AFB1 combined with β-naphthoflavone (BNF, 1 µM), with or without a 2-hour pre-treatment with CH223191 antagonist (10 µM). Bars represent mean ± SEM (*n* = 3). Statistical comparisons between conditions with and without CH223191 pre-treatment were performed using one-way ANOVA (for the mean number of γH2AX foci) and two-way ANOVA (for the percentage of cell classes): *p* < 0.05 (*), *p* < 0.01 (**)

The mean number of γH2AX foci per nucleus **(**Fig. 4A**)** and the distribution of cells according to γH2AX foci number **(**Fig. 4B**)** were analyzed for the different conditions. As expected, CH223191 and BNF alone did not significantly affect γH2AX foci formation compared with negative control cells. CH223191 pre-treatment significantly reduced BaP-induced γH2AX foci formation, decreasing from 2.6 to 1.5 the mean number of foci per cell. Consistently, after AhR antagonist treatment, the proportion of cells exhibiting 2–5 and > 5 γH2AX foci was significantly reduced, from 13% to 14% to 4% and 7% respectively. A similar effect was observed in cells exposed to the BaP-AFB1 (1:1) mixture, with CH223191 reducing the mean number of γH2AX foci from 3.1 to 1.8 and decreasing the percentage of cells with 2–5 and > 5 foci from 8% to 4% and 18% to 8% respectively. In contrast, no significant differences were observed between AFB1-treated cells and cells pre-treated with CH223191 prior to AFB1 exposure.

To clarify the role of AhR in the mixture effect, we used BNF as a well-established AhR agonist to determine whether activation of this pathway could enhance AFB1-induced genotoxicity. Co-exposure resulted in a significant increase in γH2AX foci formation compared with AFB1 alone, with the mean number of foci per cell rising from 2.3 to 3.3 and the proportion of cells with > 5 foci increasing from 9% to 12%, indicating that AhR activation by BNF exacerbates AFB1-induced DNA damage. This potentiation was significantly attenuated by CH223191 AhR antagonist, which reduced the mean number of γH2AX foci to 2.7 and decreased the percentage of highly damaged cells (> 5 foci) to 7.5%. Together, these findings demonstrate that the observed exacerbation of AFB1-induced genotoxicity is mediated by AhR signaling.

**Fig. 5:**
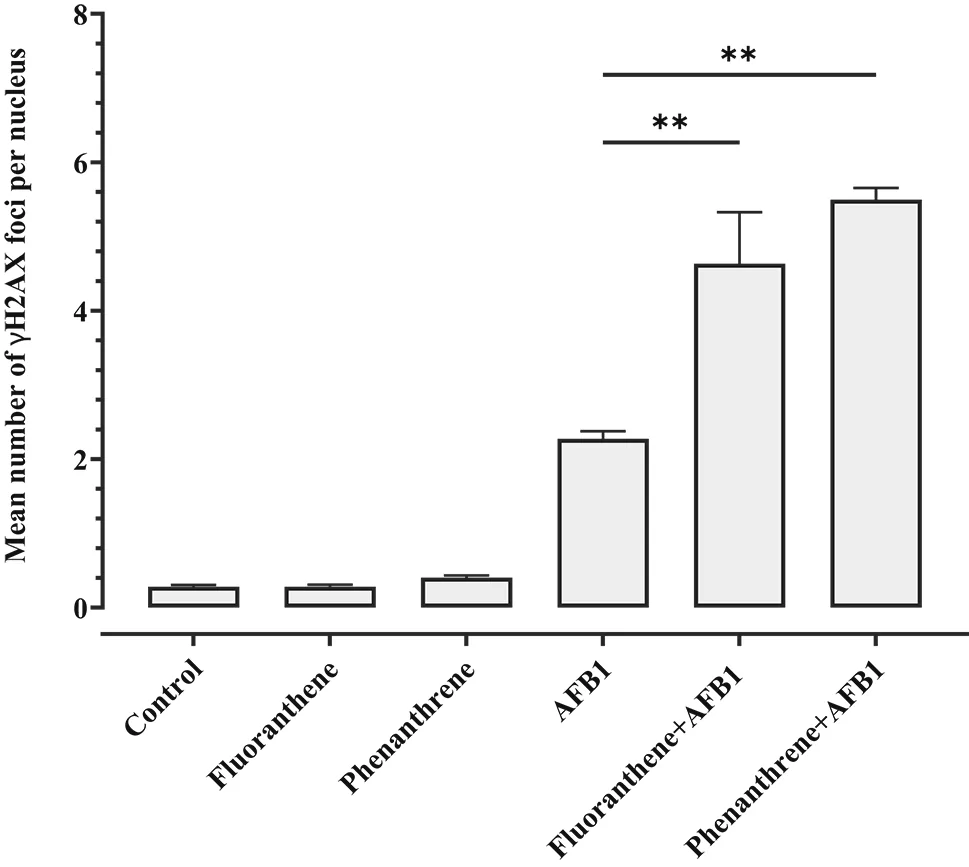
γH2AX immunofluorescence analysis after AFB1 treatment in mixture with non genotoxic PAHs. Mean number of γH2AX foci per nucleus following 24-hour treatment with Fluoranthene (50µM) and Phenanthrene (50µM) alone or in combination with AFB1 (0.301µM). Bars represent mean ± SEM (*n* = 3). Statistical comparisons between conditions were performed using one-way ANOVA: *p* < 0.05 (*), *p* < 0.01 (**)

Having established that BNF, a potent AhR agonist, exacerbates AFB1-induced genotoxicity, we next sought to determine whether AhR could be activated by other compounds. In the context of dietary co-exposure to PAH mixtures, we focused on non-genotoxic PAHs such as fluoranthene and phenanthrene which are known to activate AhR signaling. We therefore evaluated their effects, alone or in combination with AFB1, by quantifying a biomarker of DNA damage. The mean number of γH2AX foci per nucleus **(**Fig. 5**)** was analyzed under the different conditions. Although fluoranthene and phenanthrene alone did not significantly induce γH2AX foci formation compared with control cells, co-exposure with AFB1 led to a marked increase in γH2AX foci formation compared to AFB1 alone, with the mean number of foci per cell rising from 2.2 to 4.6 and 5.5 for fluoranthene and phenanthrene respectively. These results indicate that non-genotoxic AhR-activating PAHs can also exacerbate AFB1-induced DNA damage, supporting an AhR-dependent mechanism.

**Fig. 6:**
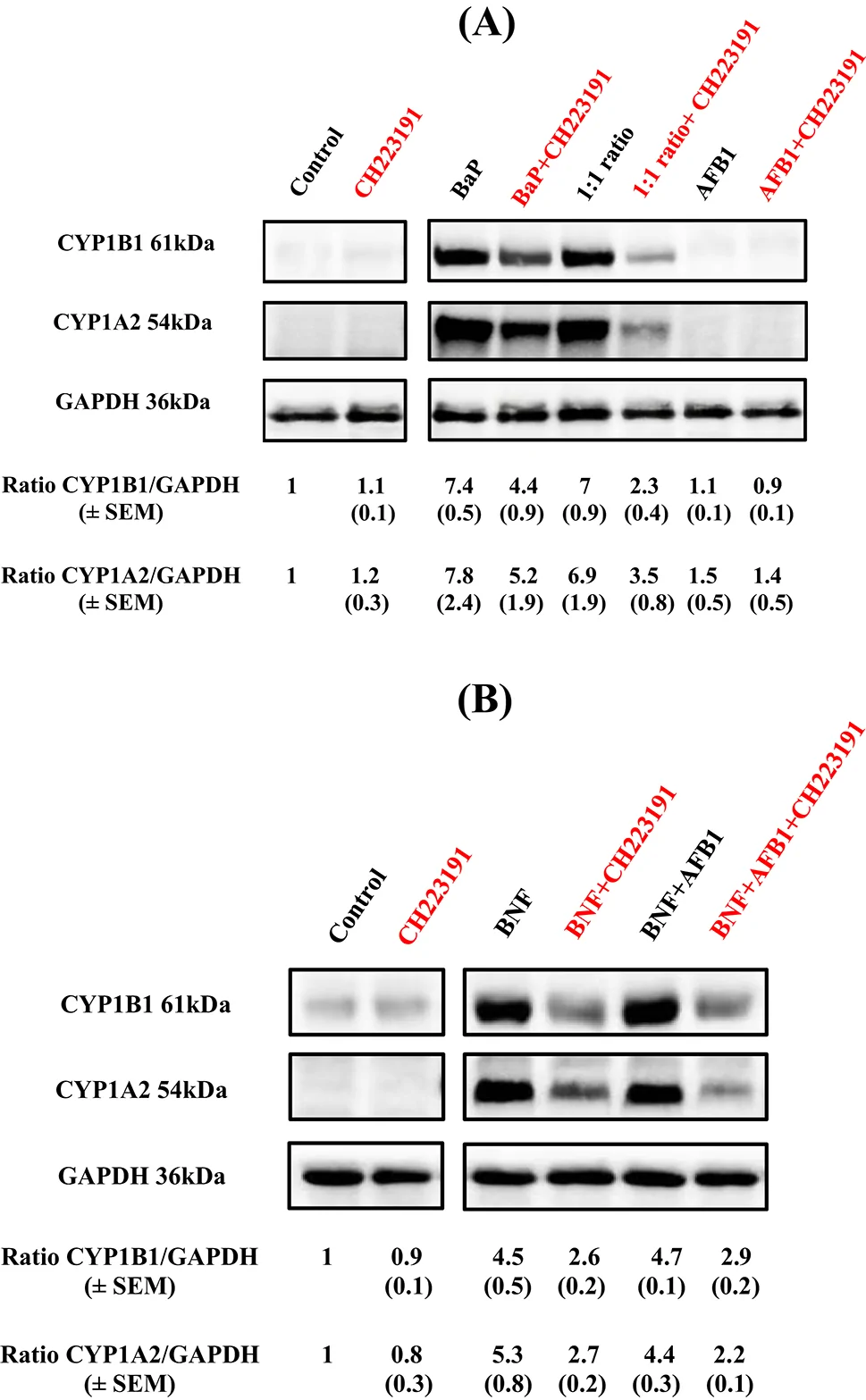
CYP1B1 and CYP1A2 protein expression after 8 h exposure. Cells were treated with BaP and AFB1 individually or as a 1:1 mixture at a BMR300% (A), or with BNF (1µM) and AFB1 (0.301µM), alone or in combination, with or without 2-hour pre-treatment with CH223191 (10µM). Protein levels were normalized to GAPDH and expressed as CYP450/GAPDH ratios. Data represent mean ± SEM of three independent experiments

To further examine the involvement of AhR in BaP-AFB1 interactions, CYP1A2 and CYP1B1 protein levels were analyzed by western blot 8 h after exposure to BaP, AFB1, or their 1:1 mixture at BMC300. Cells were also treated with 1 µM BNF agonist alone or in combination with AFB1 as controls, and all treatment conditions were performed with or without a 2-h pre-incubation with CH223191 antagonist. Treatment with CH223191 alone did not significantly alter CYP1B1 or CYP1A2 protein expression (Fig. 6A and B). Exposure to BaP and the 1:1 BaP–AFB1 mixture (BMC300) markedly induced CYP1B1 levels (7.4 and 7-fold, respectively), and CYP1A2 levels (7.8 and 6.9-fold, respectively). Pre-incubation with CH223191 attenuated these inductions, reducing CYP1B1 expression to 4.4 and 2.3-fold, and CYP1A2 expression to 5.2 and 3.5-fold, respectively **(**Fig. 6A**)**. Similarly, BNF and the BNF-AFB1 combination increased CYP1B1 protein levels (4.5 and 4.7-fold, respectively) and CYP1A2 levels (5.3 and 4.4-fold, respectively). These effects were also diminished in the CH223191 pre-treatment condition, with CYP1B1 induction reduced to 2.6 and 2.9-fold and CYP1A2 to 2.7 and 2.2-fold, respectively **(**Fig. 6B**)**. In contrast, AFB1 alone did not significantly affect CYP1B1 or CYP1A2 protein expression, further underlining the role of BaP and AhR in these CYP450 modulations.

**Fig. 7:**
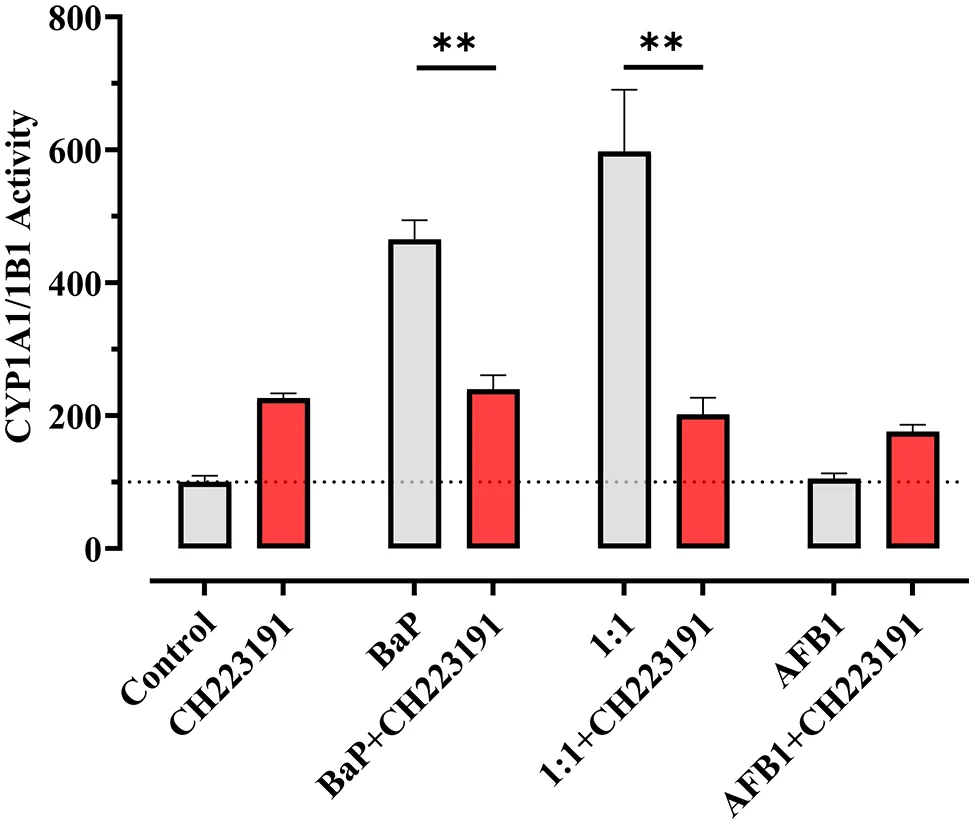
CYP1A1/1B1 activity after 8 h exposure. Cells were treated with BaP or AFB1 individually, or as a 1:1 mixture at a BMR of 300%, with or without 2-hour pre-treatment with the AhR antagonist CH223191 (10µM). Data are presented as mean ± SEM (*n* = 3). Statistical comparisons between conditions with and without CH223191 were performed using one-way ANOVA: *p* < 0.05 (*), *p* < 0.01 (**)

Finally, CYP1A1/1B1 enzymatic activities were measured to determine whether changes in protein level correlated with an altered function. BaP and the BaP–AFB1 mixture significantly increased CYP1A1/1B1 activities compared to control, with 4.7 and 5.9-fold inductions respectively **(**Fig. 7**)**. CH223191 pre-incubation significantly attenuated CYP1A1/1B1 induction, greatly reducing activity by 49% and 66.4% in BaP and BaP–AFB1-treated cells, respectively. No significant changes in CYP1A1/B1 activities were observed in AFB1-treated cells, regardless of CH223191 pre-treatment. Finally, as the mRNA level of CYP1A1 in differentiated HepaRP cells is low compared to CYP1A2 mRNA level, and given that the protein level does not seem modulated after AhR antagonist or agonist treatment (Supplementary Information 2), this suggests that CYP1A1 do not participate to the observed effect.

Collectively, these findings indicate that BaP increases CYP1B1 and CYP1A2 protein expression, as well as the CYP1A1/1B1 enzymatic activities in an AhR-dependent manner, with CYP1B1 playing a major role in addition to CYP1A2. Moreover, the results also support a key function for AhR signaling in mediating the synergistic genotoxic effects observed following co-exposure to BaP and AFB1.

## Discussion

In this study, we evaluated the applicability and relevance of the benchmark concentration (BMC) covariate approach (Sanders et al. 2025) to γH2AX genotoxic assay, aiming to characterize the BaP and AFB1 effects under co-exposure conditions. Both BaP and AFB1 are well-known genotoxic carcinogens, whose DNA-damaging effects depend on metabolic bioactivation (Smela et al. 2002; Šulc et al. 2016). To address this requirement, experiments were conducted using HepaRG-derived cells (HepaRP), an hepatic metabolically competent in vitro cell model expressing major phase I and phase II drug-metabolizing enzymes as well as key nuclear receptors, including aryl hydrocarbon receptor (AhR) (Brun et al. 2023). In contrast to differentiated HepaRG cells, comprising a mixed population of hepatocyte- and cholangiocyte-like cells, differentiated HepaRP consist exclusively of hepatocytes, providing a more homogeneous and mechanistically relevant system for investigating metabolism-dependent genotoxic interactions (Recoules et al. 2024). Successful application of the BMC covariate approach requires a clearly defined concentration–response relationship and an adequate number of biological replicates (EFSA 2017). In the present study, both compounds exhibited robust concentration-dependent increases in the mean number of γH2AX foci, fulfilling these prerequisites. These observations are consistent with previous studies reporting concentration-related increases in γH2AX immunofluorescence signal in HepaRG cells after 24 h exposure to BaP or AFB1 at comparable concentration ranges, with limited cytotoxicity (< 25%) (Quesnot et al. 2015). The selection of benchmark response (BMR) levels depends on both the genotoxic agent and the assay employed (Beal et al. 2024). Here, BMRs of 300% and 400% were chosen to represent moderate to high genotoxic responses with limited cytotoxicity.

Our results demonstrate a synergistic effect of the BaP-AFB1 mixture, as evidenced by an increased mean number of γH2AX foci under co-exposure conditions compared with exposure to each compound alone. In a previous study, co-exposure of intestinal cells to BaP and the aromatic amine 2-Amino-1-methyl-6-phenylimidazo[4,5-b]pyridine (PhIP) enhanced the formation of dG-C8-PhIP DNA adducts, although no mixture effect was observed when γH2AX was assessed using intensity-based in-cell western assay (Jamin et al. 2013). Here our study clearly demonstrated synergism when DNA damage was evaluated by quantifying γH2AX foci at the single-cell level. This discrepancy likely reflects methodological differences, as γH2AX foci number quantification is a more sensitive and biologically relevant endpoint for detecting DNA damage than global intensity measurements (Recoules et al. 2025).

After analyzing mixture data on PROAST dose-addition model in R, experimental data of BaP-AFB1 mixtures derived from the experimental points displayed clear up- and leftward deviations from the predicted additive curve. When interpretation of mixture effects is ambiguous, a complementary quantitative assessment can be applied by comparing the relative potency factor (RPF) estimates obtained with and without inclusion of mixture data in PROAST, using the ratio of overlap metric (Zoupa et al. 2020; Van Oostrom et al. 2020). In the present study, the mixture responses were unambiguous and additional quantitative analyses were therefore unnecessary.

The BMC covariate approach is a robust and increasingly recognized method for mixture toxicity assessment. In toxicological applications, this method has empirically demonstrated additive effects for both genotoxic and non-genotoxic substances (Kienhuis et al. 2015; Sanders et al. 2025), although empirical demonstrations of synergism or antagonism are scarce. Using simulated datasets, BMC covariate method has been shown to theoretically identify synergistic and antagonistic interactions (Lasch et al. 2020). In the present study, we provide experimental evidence that this method allows detecting synergistic effects between two genotoxic compounds sharing a similar mode of action, which would be expected a priori to act additively. This finding highlights the importance to not systematically assume additivity for mixture effects in regulatory risk assessment.

To understand the BaP-AFB1 mixture effect, we investigated the mechanistic basis underlying the observed synergistic interaction. In particular, we questioned the role of AhR signaling pathway and downstream CYP1 family enzymes, which are central for BaP and AFB1 metabolic bioactivations (Bonomo et al. 2017; Bukowska et al. 2022). BaP is a well-established activator of the canonical AhR signaling pathway (Kwong et al. 2024), inducing the transcriptional upregulation of CYP1A1 and CYP1B1, responsible of the BaP bioactivation into BPDE (Shimada and Fujii-Kuriyama 2004). In addition, CYP1A2 plays a key role, alongside with CYP3A4, in AFB1 bioactivation into its mutagenic epoxide (Smela et al. 2002). These observations underscore the critical importance of cytochrome P450 enzymes in mixture studies, particularly when the chemicals involved require metabolic activation to exert their toxic effects. Previously, the enhanced formation of dG-C8-PhIP DNA adducts following combined exposure to BaP and PhIP was shown to depend on CYP1-family enzymes (Jamin et al. 2013), underscoring their central role in driving the synergistic genotoxic interaction through metabolic activation. Moreover, deoxynivalenol, a well-known ribotoxic mycotoxin, has been reported to reduce the protein levels and enzymatic activities of CYP1A2 and CYP3A4, key enzymes required for AFB1 bioactivation, thereby attenuating its genotoxic effects (Willoquet et al. 2024). Based on these findings, we hypothesized that BaP-induced AhR activation could enhance CYP1A2 expression, thereby increasing the AFB1 metabolic activation and contributing to the synergistic genotoxicity observed.

Mechanistic modulation of the AhR pathway using the non-genotoxic agonist β-naphthoflavone (BNF) (Carver and Bradfield 1997) and the selective antagonist CH223191 (Zhao et al. 2010) provided critical insight into the molecular basis of BaP-AFB1 synergistic genotoxicity. BaP acts as upstream activator of AhR signaling, rapidly inducing transcriptional upregulation of CYP1A1, CYP1A2 and CYP1B1 within the first 4-hours (Krusekopf et al. 2003), which translates into elevated protein levels and enhanced CYP1A1/1B1 enzymatic activity as observed in this study. This increase in CYP1-mediated bioactivation may facilitate the conversion of both BaP and AFB1 into highly reactive electrophilic metabolites, as evidenced here by elevated DNA damage observed through γH2AX foci formation. The cumulative generation of these metabolites over time provides a mechanistic explanation for the observed synergistic effect. Pharmacological modulation of AhR confirmed this mechanism, with CH223191 pre-treatment reducing CYP1A2 and CYP1B1 protein levels and CYP1A1/1B1 enzymatic activities in both BaP and BaP-AFB1 conditions. This reduction also correlated with fewer γH2AX foci, indicating that blocking AhR limits the formation of genotoxic metabolites and mitigates DNA damage. Conversely, BNF agonist while non-genotoxic by itself, amplified AFB1-induced γH2AX foci formation, likely through enhanced CYP1 expression leading to increased generation of the reactive AFB1-8,9-epoxide. Again, this exacerbation was attenuated by CH223191, further confirming the central role of AhR-mediated CYP1 regulation in cross-bioactivating AFB1 and driving exacerbated DNA damage. Finally, as non-genotoxic polycyclic aromatic hydrocarbons (PAHs) like fluoranthene and phenanthrene enhanced AFB1 genotoxicity, this study raises concerns regarding the potential health effects of common food-contaminants co-exposure with genotoxic compounds (Bauer et al. 2018; Bramatti et al. 2023). AFB1 being one of the most potent natural carcinogen with a widespread contamination (Syraji et al. 2025), and considering highly plausible co-exposure scenarios involving AhR metabolic bioactivation, this should be taken into account to improve risk assessment and gain knowledge in the context of food toxicology.

## C**onclusion**

This study documents the synergistic BaP-AFB1 interaction in vitro and underlines the importance of AhR-dependent metabolic activation in this process. Integrating benchmark concentration (BMC) covariate modeling, this approach enabled both the quantification of the genotoxic effect and the comparison of different mixtures, finally highlighting the synergy. Characterization of the underlying mechanism reinforces the biological relevance of the observed effects. As AFB1 genotoxicity is enhanced by non-genotoxic contaminants, this raises concerns about the potential health effects of its co-exposure to common food contaminants. Considering the plausible co-exposure scenarios involving AhR metabolic bioactivation, these should be taken into account to improve risk assessment and further the understanding of food toxicology. Extending this type of study to different contaminant combinations will therefore be important for future advances in risk assessment.

## Supplementary Information

Below is the link to the electronic supplementary material.

Supplementary Material 1

## Data Availability

No datasets were generated or analysed during the current study.
