# Supplementary material for: AhR activation triggers the synergistic genotoxicity between benzo[a]pyrene and aflatoxin B1 in metabolic-competent HepaRP cells
Source: Arch Toxicol. 2026 Jul 14;100(11):5279–91. doi: 10.1007/s00204-026-04491-z (PMC13694325; doi:10.1007/s00204-026-04491-z)
Supplement: Supplementary file 1 [file 204_2026_4491_MOESM1_ESM.docx]

**BMR 400%**

**BMR 300%**

**Supplementary Information 1:** Results of the benchmark concentration (BMC) covariate analyses were obtained using the PROAST web tool version 71.1 (<https://proastweb.rivm.nl/>) at 300% and 400% benchmark response (BMR) levels, based on concentration–response datasets for benzo[a]pyrene (BaP, Red curve) and aflatoxin B1 (AFB1, Black curve). The BMC values derived at these BMR levels for both BaP and AFB1 were subsequently used to define relevant mixture compositions.

**Supplementary Information 2:** **Basal and BNF-Induced CYP1A Expression in Differentiated HepaRP Cells.** Basal mRNA expression levels of **CYP1A1** and **CYP1A2** **(A)** and CYP1A1 protein level after 8h treatment with BNF (1µM) with or without a 2-hour pre-treatment with CH223191 (10µM) **(B)**.
